# The effectiveness of combined lower limb strengthening and whole-body vibration, compared to strengthening alone, for improving patient-centred outcomes in adults with COPD: A systematic review

**DOI:** 10.4102/sajp.v76i1.1412

**Published:** 2020-06-11

**Authors:** Karina Berner, Susanna C.S. Albertyn, Sujatha Dawnarain, Lauren J. Hendricks, Jodie Johnson, Almorette Landman, Marlette Burger

**Affiliations:** 1 Division of Physiotherapy, Department of Health and Rehabilitation Sciences, Faculty of Medicine and Health Sciences, Stellenbosch University, Cape Town, South Africa

**Keywords:** chronic obstructive pulmonary disease, whole-body vibration training, strength training, pulmonary rehabilitation, lower limb muscle strength, quality of life, functional exercise capacity, pulmonary function

## Abstract

**Background:**

People with chronic obstructive pulmonary disease (COPD) experience various impairments, reducing quality of life (QoL). Rehabilitation that does not elicit dyspnoea, such as strength training, is recommended to improve patient outcomes.

**Objectives:**

To systematically review evidence for the effectiveness of lower limb strengthening combined with whole-body vibration training (WBVT), versus lower limb strengthening alone, in adults with COPD for improving lower limb muscle strength, functional exercise capacity (FEC), pulmonary function and QoL.

**Method:**

Eight databases were searched (inception–May 2019). Only randomised controlled trials (RCTs) with PEDro scores ≥ 5/10 were included. Heterogeneity rendered meta-analyses inappropriate; thus data were synthesised narratively.

**Results:**

Five RCTs (mean PEDro score: 5.8/10) were included. Only one RCT showed a significant difference for leg press peak force (kg) at 12 weeks (*p* = 0.001), favouring WBVT. FEC improved significantly (*p* < 0.05) in favour of WBVT at 3 and 12 weeks. Combined training was not more effective for short-term (≤ 12 weeks) improvements in pulmonary function or QoL.

**Conclusion:**

Level II evidence suggests that combining strengthening with WBVT has significant beneficial short-term effects on FEC in adults with COPD. Results are limited by the small number of studies and small sample sizes. Combined WBVT and strengthening was not more effective than strengthening alone for improving lower limb muscle strength, pulmonary function and QoL.

**Clinical implications:**

Combining low (6–10 Hz) to moderate (24–26 Hz) frequency WBVT with strengthening may be a more effective modality to improve FEC than strengthening alone, should resources allow.

## Introduction

Chronic obstructive pulmonary disease (COPD) is a common, incurable but manageable disease. Characteristic to the condition is airway and/or alveolar abnormalities, leading to chronic respiratory symptoms and limited airflow that is often triggered by exposure to toxic gases (Mirza et al. 2018; Vogelmeier et al. 2017). The presentation is often a slow, progressive onset of symptoms in people older than 40 (Viviers & Van Zyl-Smit 2015). The Global Burden of Disease Study reported a prevalence of 251 million COPD cases globally in 2016 (World Health Organization 2017). Despite lacking information regarding COPD prevalence in South Africa, it is predicted that by 2020 the disease will become one of the three leading causes of death attributed to non-communicable disease globally (Viviers & Van Zyl-Smit 2015).

South Africans are exposed to COPD risk factors such as cigarette smoke, inorganic dust, indoor use of biomass fuel for heating and cooking and HIV (World Health Organization 2017). Symptoms include a cough, sputum production and progressive worsening of shortness of breath. Functional tasks such as walking or stair climbing lead to air trapping and increased hyperinflation, exacerbating breathlessness (Khachi, Barnes & Antoniou 2010; O’Donnell et al. 2007; Viviers & Van Zyl-Smit 2015). This increased breathlessness incites anxiety, which leads to more breathlessness, causing a chain reaction where physical activities are avoided (O’Donnell et al. 2007).

Common systemic presentations in COPD linked to increased mortality include impairments in respiratory and peripheral muscle function and mass (Barreiro & Gea 2015). Such dysfunction is heterogeneously distributed among muscle groups; however, the strength of upper limbs and respiratory muscles is better preserved than that of lower limbs (Maltais et al. 2014). People with COPD struggle to participate in physical activity (Ek & Ternestedt 2008), mainly because of dyspnoea and leg fatigue (Maltais et al. 2014). This further reduces exercise tolerance and quality of life (QoL) and increases healthcare utilisation (Barreiro & Gea 2015).

Chronic obstructive pulmonary disease has very few treatment options (pharmacological or non-pharmacological) that influence mortality (Keen & Medarov 2017). Pulmonary rehabilitation is effective for improving exercise tolerance, dyspnoea and fatigue (World Health Organization 2018). As lower limb dysfunction commonly occurs in people with COPD who are struggling with severe dyspnoea (Vonbank et al. 2012), international guidelines recommend combining endurance and strength training as a part of pulmonary rehabilitation (Iepsen et al. 2015; Spielmanns et al. 2017a; World Health Organization 2018). Strength training has a decreased cardiovascular response compared to endurance exercise. The added benefit of strength training is that it has a lower level of oxygen consumption and minute ventilation, resulting in decreased dyspnoea during training. Strength training is thus considered an effective and feasible treatment option in the clinical setting for people with COPD, although optimal training strategies and mechanisms of improvement are still under investigation (Daabis, Hassan & Zidan 2017).

A proposed complementary modality to strength training in people with COPD is whole-body vibration training (WBVT) (Gloeckl, Heinzelmann & Kenn 2015). The WBVT is conducted on a vibrating platform moving in sinusoidal oscillations and reportedly improves muscle activation when implemented simultaneously with static and dynamic exercises (Spielmanns et al. 2017a). Strength training incorporating WBVT is reported to be a safe and time-saving intervention that may be more effective than strength training alone to result in improved exercise performance and other person-centred outcomes in people with COPD (Spielmanns et al. 2017a). Vibrations are applied at different frequencies, which cause the muscle relaxation period to be interrupted. This contributes to higher output in muscle power (Spielmanns et al. 2017a) and there is some evidence that WBVT effectively improves lower limb strength in people with COPD (Koczulla et al. 2014).

Four systematic reviews (Cardim et al. 2016; Gloeckl et al. 2015; Yang et al. 2016; Zhou et al. 2018) have been published dealing with the effect of WBVT on pulmonary function, functional exercise capacity (FEC) and QoL in people with COPD. The most recent review (Zhou et al. 2018) showed that WBVT was beneficial for improving FEC, but limited evidence was found regarding the effects on pulmonary function and QoL. Only two systematic reviews (Cardim et al. 2016; Gloeckl et al. 2015) assessed muscle strength as an outcome in people with COPD and found no difference between the WBVT and control groups. However, these reviews based their findings on the same single randomised controlled trial (RCT) (Pleguezuelos et al. 2013). The most recent systematic review (Zhou et al. 2018) did not assess muscle strength. Since the publication of the three systematic reviews up to 2016, more RCTs have been published, and include lower limb muscle strength as an outcome. Therefore, the purpose of this systematic review was to update the current evidence regarding the effect of WBVT on muscle strength, FEC, pulmonary function and QoL in people with COPD.

## Methodology

The Preferred Reporting Items for Systematic Reviews and Meta-Analyses (PRISMA) guidelines were used to conduct this systematic review (Liberati et al. 2009).

### Search strategy

Eight computerised bibliographic databases (PubMed, Cochrane Library, Science Direct, EBSCOhost: CINAHL, EBSCOhost: SPORTDiscus, PEDro, Scopus and ProQuest Medical Library) were accessed and searched via Stellenbosch University library services. Date limits were set from inception to May 2019. Keywords included the following: ‘chronic obstructive pulmonary disease’, ‘chronic obstructive airway disease’, ‘chronic obstructive lung disease’, ‘chronic airflow obstruction*’, ‘whole body vibration’, rehabilitation, ‘pulmonary rehabilitation’, exercise, ‘resistance training’ and strength*. The databases were divided among the research team, with two authors assigned to each database. Author pairs independently searched their allocated databases, using the same search strategy. Each step of the search process was documented and cross-checked. The authors independently reviewed titles, abstracts and full-texts according to pre-determined eligibility criteria (as discussed below). Articles selected for potential inclusion were compared and disagreements were discussed until consensus was reached.

### Study eligibility criteria

#### Type of studies

Only published RCTs available in English and scoring at least 5/10 on the PEDro scale (Verhagen et al. 1998) were included. According to the National Health and Medical Research Council (NHMRC) Hierarchy of Evidence (Coleman et al. 2009), level II evidence is best suited to answer intervention (effectiveness) questions in systematic reviews, whereas levels III–IV become progressively less reliable.

#### Type of participants

Study participants could include men and women older than 40 years with a clinical diagnosis of COPD (stages I, II, III or IV according to the Global Initiative for Chronic Obstructive Lung Disease [GOLD] classification [Han et al. 2013]). Randomised controlled trials with participants suffering from conditions that may affect performance or safety of lower limb strength exercises (musculoskeletal comorbidities; COPD exacerbation in the four weeks prior to study participation) were excluded.

#### Type of interventions

Studies in which participants received WBVT combined with lower limb strength training at low (6 Hz – 10 Hz), moderate (24 Hz – 26 Hz) or high frequencies (≥ 35 Hz) were included.

#### Types of comparisons

Studies in which participants received lower limb strength training alone were included.

#### Type of outcomes

Studies that measured at least one of the following outcomes were included: lower limb muscle strength, FEC, pulmonary function and QoL.

### Methodological appraisal

Articles were methodologically appraised using the PEDro scale, a valid and reliable assessment of the methodological quality of clinical trials (Verhagen et al. 1998). The scale appraises internal validity and statistical reporting according to 10 criteria (De Morton 2009). Two authors were assigned to each article and independently scored the articles. Scores were compared and if there were discrepancies, a third author within the group was asked to appraise the article. If consensus was still not reached, the senior author (M.B.) was consulted to determine the final score.

### Data extraction and analysis

Data were extracted using the adapted Joanna Briggs Institute (JBI) Data Extraction Form for Systematic Review of Experimental Studies (Pearson, Field & Jordan 2009). Articles were divided among the research team and each author was responsible for extracting data according to their assigned sections. After collating the information, findings were compared and a consensus was reached that all data were complete and correct. A critical analysis of the data was then conducted. Authors worked in pairs, each analysing different outcomes independently, whereafter findings were compared. If a pair’s results differed, the data were presented to the rest of the group for discussion and consensus. If consensus was not reached, the senior author (M.B.) was contacted to make the final decision. Because of heterogeneity in the data, statistical pooling via meta-analysis was deemed inappropriate. Data from the various studies were thus summarised narratively based on the review objectives.

### Ethical consideration

This study consists of secondary research; thus, ethical approval was not required for this systematic review.

## Results

### Search results

**FIGURE 1:**
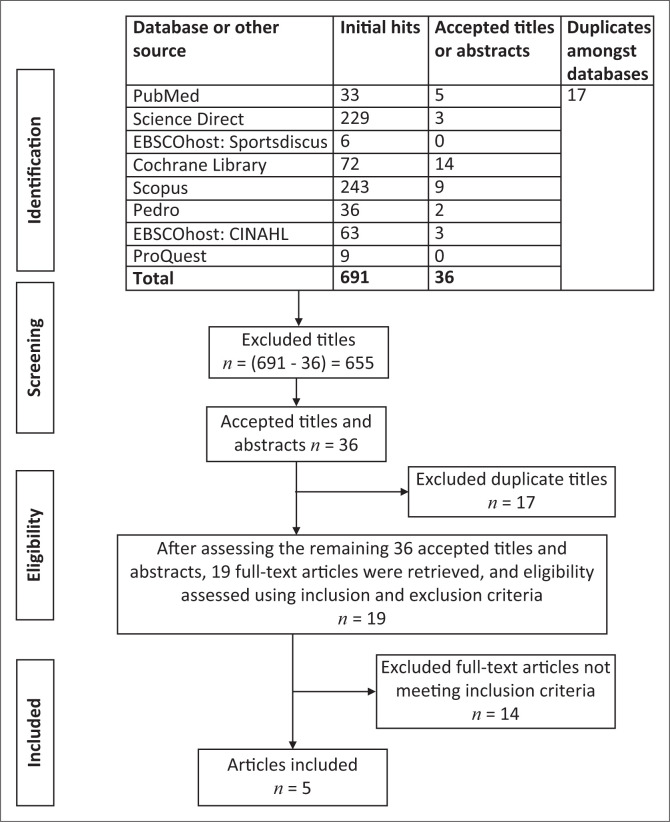
Search results.

The search yielded 691 initial hits, of which 655 irrelevant titles were removed. Of the 36 remaining titles and abstracts, 17 duplicates were excluded. Subsequently, 19 full-texts were assessed according to the review’s eligibility criteria. Fourteen full-texts did not meet these criteria, leaving five articles suitable for analysis (Figure 1).

### Methodological appraisal

The methodological quality scores of the five included RCTs (Gloeckl et al. 2012, 2017; Salhi et al. 2015; Spielmanns et al. 2017a, 2017b) ranged from 5/10 to 7/10 on the PEDro scale, with a mean score of 5.8/10.

**FIGURE 2:**
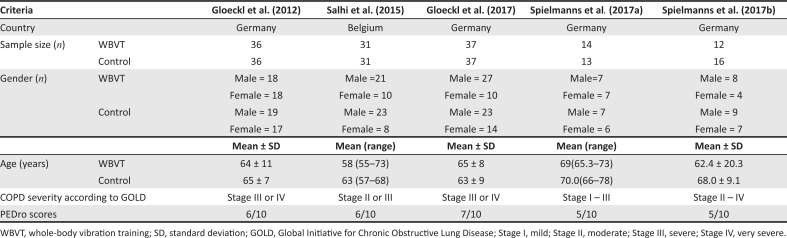
Description of included studies.

Criteria 5 and 6 (blinding of participants and therapists) were not met in any of the studies (Figure 2).

### Description of study sample

Most of the studies had small sample sizes, contributing to a total sample size of 263 (130 and 133 participants in the intervention and control groups, respectively). Studies included predominantly more men than women. Mean participant ages ranged from 58 to 70 years. All participants were diagnosed with COPD (stages I–IV) and were otherwise healthy. According to the World Bank classification (World Bank Group 2019), all five studies were conducted in high-income countries (Table 1).

**TABLE 1:** Description of interventions.

	Intervention: WBVT combined with lower limb strength training			Control: Lower limb strength training alone		
	Intervention method	Total duration	Dosages and frequency	Intervention method	Total duration	Dosages and frequency
**Gloeckl et al. (2012)**	Supervised dynamic squatting regime (squatting position: not specified) Exercise training (endurance, strength training for major muscle groups) Multi-disciplinary inpatient rehabilitation programme (medical care, breathing therapy education, nutritional counselling, psychological support)	3 weeks Squat training: 3 days per week Rehabilitation and exercise: 5 days per week	**Squat exercises:** Surface: Side-alternating WBV plate Sets: 3 × 3 min Repetitions/time: self-paced over 3 min (9 min total exposure time) **WBVT settings:** Frequency: 24 Hz–26 Hz Amplitude: 6 mm **Endurance training:** 15 min cycling at 60% peak Watt **Strength training**: 4–6 exercises of major muscle groups Sets: 3 Repetitions: 20 Intensity: 20 repetition max Multidisciplinary inpatient rehabilitation program: N/A (standard inpatient care)	Supervised dynamic squatting regime (squatting position: not specified) Multi-disciplinary inpatient rehabilitation program (medical care, breathing therapy education, nutritional counselling, psychological support) Exercise training (endurance, strength training for major muscle groups)	3 weeks Squat training: 3 days per week Rehabilitation and exercise training: 5 days per week	**Squat exercises:** Surface: Normal flat surface (floor) Sets: 3 × 3 min Repetitions/time: self-paced over 3 min (9 min total exposure time) **Endurance training:** 15 min cycling at 60% peak Watt **Strength training:** 4–6 exercises major muscle groups Sets: 3 Repetitions: 20 Intensity: 20 repetition max Multidisciplinary inpatient rehabilitation program: N/A (standard inpatient care)
**Salhi et al. (2015)**	Supervised static and dynamic squatting regime;Three squatting positions: High squat (knee angle 120°–130°) Deep squat (knee angle 90°) Wide-stance squat Fourth lower body exercise: Lunge Four upper body exercises:Front raiseBent over lateralBicep curlCross over Exercise training also included endurance training (preceding the strength training) Oxygen prescribed to patients who desaturated <88% during training	12 weeks 3 days per week	Initial training volume and intensity was low but increased progressively in line with overload principle over 12 weeks. After 6 weeks static exercises were replaced by dynamic exercises **Squat exercises:** Surface: vertical WBV plate Sets: increasing 1–3 (over 12 weeks) Repetitions/time: 30 s to 1 min Rest: shortening rest period over 12 weeks **WBVT settings:** Frequency: 27 Hz (unchanged) Amplitude: peak-to-peak 2 mm (unchanged) **Endurance training:** 15 min (prior to strength training)	No squatting exercises specifically mentioned. **Exercise training:** Conventional resistance training regime; Multigym equipment used for muscle training (quadriceps, hamstrings, deltoids, biceps brachii, triceps brachii, pectorals) Exercise training also included endurance training (preceding the strength training) Oxygen prescribed to patients who desaturated <88% during training.	12 weeks 3 days per week	Exercise intensity and repetitions were progressively increased over 12 weeks. **Exercise training:** Strength training: (patient started) Sets: 3 Reps: 10 (first week) Intensity: 70% of initial one-repetition maximum (IRM) of 10 reps in first week. Intensity and number of repetitions were increased over time to maintain Borg score of between 4 and 6 in each exercise for both dyspnoea & fatigue. After 6 weeks, resistance levels were adjusted based on results from new IRM force tests **Endurance training:** 15 min (prior to strength training)
**Gloeckl et al. (2017)**	Supervised dynamic squatting regime (squatting position: knee and hip flexion between 90° and 120° without holding on to anything) Exercise training consisted of endurance and strength training for major muscle groups. Multi-disciplinary inpatient pulmonary rehabilitation program: medical carebreathing therapy educationnutritional counsellingpsychological counselling	3 weeks 5 days per week Pulmonary rehabilitation: standard duration (Germany)	**Squat exercises:** Surface: side-alternating WBV plate Sets: 4 Repetitions/time: 2 min Intensity: body weight (self-paced) **WBVT settings:** Frequency: 24 Hz–26 Hz Amplitude: 5 mm peak-to-peak **Endurance training:** 15 min cycling at 60% peak Watt **Strength training:** 4–6 exercises major muscle groups on gym machines Sets: 3 Repetitions: 15–20 Intensity: Maximum load to tolerate repetitions required to momentary muscle failure	Supervised dynamic squatting regime (squatting position: knee and hip flexion between 90° and 120° without holding on to anything) Exercise training consisted of endurance and strength training for major muscle groups. Multi-disciplinary inpatient pulmonary rehabilitation programme: medical care breathing therapy education nutritional counselling psychological counselling	3 weeks 5 days per week Pulmonary rehabilitation: standard duration (Germany)	**Squat exercises:** Surface: normal flat surface (floor) Sets: 4 Repetitions/time: 2 min Intensity: body weight (self-paced) **Endurance training:** 15 min cycling @ 60% peak Watt **Strength training:** 4–6 exercises major muscle groups on gym machines Sets: 3 Repetitions: 15–20 Intensity: maximum load to tolerate repetitions required to momentary muscle failure
**Spielmanns et al. (2017a)**	Supervised static squatting regime (squat position: knees bent at more or less 150°. Arms by side or on the arm rest of the device) Exercise training also included endurance training and low-intensity stretching (warm up/cool-down)	12 weeks 2 days per week (30 min sessions)	**Squat exercises:** (total time of 15 min per squat session) Surface: side-alternating WBV plate Frequency: 6 Hz–10 Hz (week 1–4); 12 Hz–18 Hz (weeks 5–8); 21 Hz–24 Hz (week 9–12) Amplitude: 4–6 mm peak-to-peak (week 1–12) Sets: 3 (week 1–12) Reps/duration: 2 min (week 1–4); 3 min (week 5–8); 3 min (week 9–12) Rest 2 min between reps – patients allowed to sit or walk in the rest periods **Endurance training:** 10-min low-intensity treadmill walking or cycling 5-min cooldown	No squatting exercises specified Calisthenics training in group training sessions, combined with relaxation and breathing retraining.	12 weeks 2 days per week (30 min sessions)	Group training sessions were a 30 min session. No details were provided in the article regarding the type of calisthenics exercises, sets or repetitions
**Spielmanns et al. (2017b)**	Dynamic squat position and sequence: knees bent at 10°–20° – followed by 30°–40° flexion, without holding on with the arms) Exercise training additionally consisted of endurance training, weight training and cardiovascular training. Training for daily living orientation also included	12 weeks 1 day per week (90 min sessions)	**Squat exercises:** Surface: side-alternating WBV plate Sets: 3 (week 1–12) Reps/duration: 20 squats **WBVT settings:** Frequency: 24 Hz–26 Hz Amplitude: 3 mm peak-to-peak **Additional strength training:** weight-training (dumbbells and TheraBand)Extensive details of the additional endurance and strengthening exercise program provided elsewhere† **Endurance/cardiovascular training:** walking, ergometer training, stair-climbing, dance etc. All sessions were 90 min	Dynamic squat position and sequence: knees bent at 10°–20° – followed by 30°–40° flexion, without holding on with the arms) Exercise training additionally consisted of endurance training, weight training and cardiovascular training. Training for daily living orientation also included	12 weeks 1 day per week (90 min sessions)	Squat exercises: Surface: floor Sets: 3 (week 1–12) Reps/duration: 20 squats **Additional strength training:** weight-training (dumbbells and TheraBand)Extensive details of the additional endurance and strengthening exercise program provided elsewhere† **Endurance/cardiovascular training:** walking, ergometer training, stair-climbing, dance etc. All sessions were 90 min

WBV, whole-body vibration; WBVT, whole-body vibration training; HZ, Hertz (unit of frequency); N/A, not applicable; IRM, initial one-repetition maximum.

† , Extensive exercise programme available in Spielmanns, Göhl, Schultz & Worth (2015).

### Description of intervention

Although all five RCTs included the performance of squatting exercises on a vibration plate as a core component of the intervention, substantial variation was evident in protocols across the studies (in terms of WBVT settings, squatting regimes and additional strength training) (Table 2).

**TABLE 2:** Outcome measures used and assessment intervals.

	Gloeckl et al. (2012)	Salhi et al. (2015)	Gloeckl et al. (2017)	Spielmanns et al. (2017a)	Spielmanns et al. (2017b)	
**Outcome measures**	6MWT; FEV1; CRDQ	Peak isometric torque (Nm); 6MWT; VO_2_ max; FEV1; CRDQ	Peak isometric knee extension (N); 6MWT	Peak leg press force (kg); 6MWT; CAT; SGRQ	6MWT; FEV1 CAT; CRDQ	Baseline
	6MWT; FEV1; CRDQ	-	Peak isometric knee extension (N); 6MWT	-	-	3 weeks
	-	Peak isometric torque (Nm); 6MWT; VO_2_ max; FEV1; CRDQ	-	Peak leg press force (kg); 6MWT; CAT; SGRQ	6MWT; FEV1 COPD assessment test; CRDQ	12 weeks

6MWT, 6-minute walk test; FEV1, forced expiratory volume; CRDQ, Chronic Respiratory Disease Questionnaire; VO_2_ max, maximal oxygen uptake; CAT, COPD assessment test; SGRQ, St George’s Respiratory Questionnaire.

### Description of outcome measures

The outcome measures used to assess lower limb muscle strength, FEC, pulmonary function and QoL along with the assessment intervals are shown in Table 3. Salhi et al. (2015) and Spielmanns et al. (2017a and 2017b) measured all outcomes at baseline and 12 weeks, while Gloeckl et al. (2012) and Gloeckl et al. (2017) measured outcomes at baseline and 3 weeks.

**TABLE 3:** Results for functional exercise capacity as measured by 6-minute walking distance at baseline and after treatment at 3 weeks (Gloeckl et al. 2012, 2017) and 12 weeks (Salhi et al. 2015; Spielmanns et al. 2017a, 2017b).

6MWD		Baseline mean ± SD or median (IQR)	After treatment median change (IQR)	Mean change ± SD	Intragroup *P*	Intergroup *P*
Gloeckl et al. (2012) (moderate frequency)	WBVT	349 ± 112 (completed rehab)	-	64.0 ± 59.1	-	0.046* (3 weeks)
	Control	344 ± 104	-	37.3 ± 52.2	-	-
Salhi et al. (2015) (moderate frequency)	WBVT	420 (330–480)	453 (389–519)	-	0.003*	0.070 (12 weeks)
	Control	430 (327–511)	495 (425–555)	-	0.001*	-
Gloeckl et al. (2017) (moderate frequency)	WBVT	335 ± 107	55 (43–69)	-	< 0.001*	< 0.001* (3 weeks)
	Control	350 ± 104	32 (19–45)	-	< 0.001*	-
Spielmanns et al. (2017a) (low frequency)	WBVT	507.0 (438.8–594.8)	596.0 (532–704)	-	0.001*	0.001* (12 weeks)
	Control	490.0 (410–608)	499.0 (480–629)	-	0.100	-
Spielmanns et al. (2017b) (moderate frequency)	WBVT	553 ± 7	-	561 ± 9	0.574	0.798 (12 weeks)
	Control	481 ± 121	-	490 ± 125	0.484	-

SD, standard deviation; WBVT, whole-body vibration training.

* , Significant differences at *p* < 0.05.

### The effect of lower limb strength training combined with whole body vibration training compared to lower limb strength training alone on review outcomes

The effect of lower limb strength training combined with WBVT, compared to lower limb strength training alone, is presented in the following paragraphs under the headings ‘lower limb muscle strength’, ‘FEC’, ‘pulmonary function’ and ‘QoL’.

The group receiving strength training combined with WBVT will from here on be referred to as only ‘WBVT’, and the group receiving lower limb strength training alone as the ‘control’.

#### Lower limb muscle strength

Knee extension peak isometric torque (Nm) improved significantly within the control group only in the study conducted by Salhi et al. (2015) (baseline median 105 Nm, interquartile range [IQR] 90 Nm –122 Nm; 12-week median 116 Nm, IQR 100 NM – 162 Nm; *p* = 0.009). No significant change was found within the WBVT group (baseline median 92 Nm, IQR 70 Nm – 114 Nm; 12-week median 96 Nm, IQR 72 Nm – 140 Nm; *p* = 0.23) or between the WBVT and control groups (*p* = 0.07). Knee extension peak isometric force (N) improved significantly in WBVT and control groups in the study conducted by Gloeckl et al. (2017) (both *p* < 0.001). Mean changes from baseline to 3 weeks were, however, not significantly different between groups (WBVT mean change 24.7 N, 95% confidence interval [CI], 11.6 N – 37.8 N; control mean change 23.7 N, 95% CI, 10.0 N – 37.4 N, *p* = 0.912).

Leg press peak force (kg) demonstrated a significant between-group difference at 12 weeks (*p* = 0.001) in favour of the WBVT group in the work conducted by Spielmanns et al. (2017a). The WBVT group improved significantly (baseline median 92.0 kg, IQR 66.7 kg – 138 kg; 12-week median 123.0 kg, IQR 93.3 kg – 166.7 kg; *p* = 0.001), while no significant improvement occurred in the control group (baseline median 130.0 kg, IQR 112 kg – 142 kg; 12-week median 127.5 kg, IQR 115 kg – 153.8 kg; *p* = 0.44).

#### Functional exercise capacity

Three studies (Gloeckl et al. 2012, 2017; Spielmanns et al. 2017a) revealed that the 6-minute walking distance (6MWD) improved significantly more in the WBVT group compared to the control group (all *p* < 0.05). Salhi et al. (2015) observed significant improvements within both groups, but the between-group difference was non-significant (*p* = 0.07). Spielmanns et al. (2017b) found no significant difference within or between groups (*p* = 0.798) (Table 4). Salhi et al. (2015) additionally measured VO_2_ peak values and found significant improvements in both the WBVT (baseline median 1040, IQR 790–1350; 12-week median 1030, IQR 890–1550; *p* < 0.05) and control (baseline median 1020, IQR 950–1230; 12-week median 1170, IQR 1040–1370; *p* < 0.05) groups. No significant difference was found between groups after 12 weeks (*p* = 0.60).

#### Pulmonary function

Gloeckl et al. (2012) found no significant between group differences in forced expiratory volume (FEV1) at 3 weeks (WBVT mean change 0.8, SD 6.6; control mean change 2.6, SD 5.7; *p* = 0.22). Salhi et al. (2015) did not report values for FEV1 mean change or results after treatment, but reported that mean changes at 12 weeks between groups were non-significant (*p* > 0.05). Spielmanns et al. (2017b) found no significant differences in mean change at 12 weeks between groups (WBVT mean change -3.5, SD 22.5; control mean change -0.3, SD 7.7; *p* = 0.944).

In addition, neither the WBVT (baseline mean 48.4, SD 20.9; 12-week mean 44.9, SD 18.9; *p* = 0.753) nor control (baseline mean 51.2, SD 17.3; 12-week mean 50.9, SD 16.4; *p* = 0.683) group showed a significant difference in FEV1 after 12 weeks.

#### Quality of life

Studies reporting on the Chronic Respiratory Disease Questionnaire (CRDQ) presented results as total score and/or individual domains. Gloeckl et al. (2012) found no significant differences at 3 weeks between groups in total score (WBVT mean change 3.09, SD 4.28; control mean change 2.92, SD 3.13; *p* = 0.86) or any individual domains (all *p* > 0.05). In the work conducted by Salhi et al. (2015), it was noted that total score improved significantly in both the WBVT (baseline median 72, IQR 66–96; 12-week median 92, IQR 75–99; *p* < 0.05) and control groups (baseline median 75, IQR 63–91; 12-week median 94, IQR 72–107; *p* < 0.05). No significant difference, however, existed between the groups at 12 weeks (*p* > 0.05). Results were not reported for individual domains. Spielmanns et al. (2017b) did not report results for total score, but found that the WBVT group improved significantly in the domains of fatigue (baseline mean 3.88, SD 1.25; 12-week mean 4.48, SD 1.32; *p* = 0.022), emotional function (baseline mean 4.08, SD 1.16; 12-week mean 4.92, SD 1.19; *p* = 0.007) and mastery (baseline mean 4.04, SD 1.21; 12-week mean 5.04, SD 1.40; *p* = 0.018). The control group showed no significant improvements in any individual domain (all *p* > 0.05). A significant between-group difference was noted in favour of WBVT in the emotional functioning domain at 12 weeks (*p* = 0.04).

The COPD assessment test (CAT) scores demonstrated a significant between-group difference at 12 weeks in favour of the control group (*p* = 0.02) in Spielmanns et al. (2017a). Within-group changes were non-significant for WBVT (baseline median 18.5, IQR 15–20; 12-week median 18.0, IQR 11–24; *p* = 0.14) as well as control groups (baseline median 16.0, IQR 12–21; 12-week median 14.0, IQR 10–22; *p* = 0.38). Using this same outcome measure, Spielmanns et al. (2017b) found no between-group differences at 12 weeks (*p* = 0.139). Within-group differences were non-significant for WBVT (baseline mean 20.3 ± 7.7; 12-week mean 17.8 ± 7.2; *p* = 0.321) or control (baseline mean 20.8 ± 5.5; 12-week mean 19.9 ± 7.0; *p* = 0.321) groups (Spielmanns et al. 2017b).

Using the St George Respiratory Questionnaire (SGRQ), Spielmanns et al. (2017a) found no significant score changes within the WBVT (baseline median 43.5, IQR 27.5–52.8; 12-week median 38.1, IQR 19.4–46.3; *p* = 0.08) or control groups (baseline median 43.4, IQR 25.1–52.9; 12 week median 42.5, IQR 27.6–48.7; *p* = 0.31). The between-group difference was also non-significant (*p* = 0.11).

## Discussion

This review aimed to determine the effectiveness of lower limb strength training combined with WBVT, compared to lower limb strength training alone, on various patient-centred outcomes in COPD. To our knowledge, this was the first review to focus on lower limb strength training as a comparative element. Results demonstrated limited evidence that combining strength training and WBVT may improve lower limb strength (measured as leg press strength) more effectively than strength training alone after at least 12 weeks in adults older than 40 with mild-to-moderate COPD. Results further suggest that combining strength training with WBVT improves FEC (measured as 6MWD) significantly more than strength training alone in the short term (≤ 12 weeks) for adults with mild-to-severe COPD. Similar to our review’s findings, previous systematic reviews (Cardim et al. 2016; Gloeckl et al. 2015; Yang et al. 2016; Zhou et al. 2018) revealed that WBVT had beneficial effects on FEC. Evidence was lacking or limited for pulmonary function and QoL in all these reviews.

Skeletal and lower limb muscle dysfunction is considered one of the most troublesome systemic manifestations of COPD and is characterised by limb muscle atrophy, weakness and decreased endurance (Chen et al. 2018). Although the relationship between limb muscle dysfunction and COPD is still poorly understood, the loss of type I muscle fibres, reduced oxidative enzyme activity, reduced capillary-to-fibre ratio, nutritional depletion and inflammation have been suggested as causal factors (Maltais et al. 2014). Peripheral muscle atrophy is also closely linked to increased hospitalisations and decreased life expectancy in people suffering from COPD (Swallow et al. 2007). A mid-thigh muscle cross-sectional area of < 70 cm^2^ is associated with a fourfold increase in mortality after adjusting for age, sex and lung function (FEV1) (Maltais et al. 2014). International clinical practice guidelines recommend that exercise and strength training of lower limb muscles should be a mandatory component of pulmonary rehabilitation for people with COPD to improve lower limb muscle strength, function and mass (Ries et al. 2007). Such training may prevent disease progression and improve prognosis regardless of the severity of pulmonary disease (Jaitovich & Barreiro 2018).

Across the RCTs assessing lower limb strength and included in this review, participant groups performing squats (with or without WBVT) or conventional progressive resistance programmes (even without squats and/or WBVT) demonstrated improved knee extension strength. Thus, regarding targeted knee extension strength, it seems that squatting may be effective regardless of adding WBVT. Furthermore, a conventional resistance programme may be preferable to combined squatting and WBVT. In terms of more general lower limb strength (leg press), it remains unclear whether combining squats with WBVT holds additional benefits over squatting alone because of the non-comparable training approaches noted in the RCTs. It may, for example, simply be that squatting was more effective than the calisthenics (bodyweight exercises, similar to gymnastics) employed by Spielmanns et al. (2017a). In other populations, increased muscle strength is a major benefit of WBVT, especially in the elderly (Lau et al. 2011). The current review also included a mostly elderly sample. Reasons for discrepant results may be differences in the WBVT operation protocols, exercise protocols, the specific pathology and variations in the type of squats.

Another reason for the observed results may be the peripheral muscle dysfunction associated with COPD, along with the different muscle strength assessments used in the various studies: a leg press test (a closed-chain and multi-joint or muscle movement) versus a pure knee extension test (an open-chain and single muscle or joint movement). Quadriceps weakness is a particular concern in people with COPD; the weakness becoming more prevalent with increasing disease severity (Kharbanda, Krishnan & Ramakrishna 2015). Quadriceps femoris neuromuscular activation is greater with isolated knee extension compared to a multi-joint leg press (Andersen et al. 2006). It may thus be that assessing this muscle in isolation in adults with COPD has less potential for demonstrating improvement than using a more functional assessment involving additional muscles. A leg press also more closely resembles the squatting exercises performed on the vibration plate, involving more muscles (gluteus maximus, quadriceps femoris, gastrocnemius, soleus, hamstrings and adductor muscles) than isolated knee extension (mainly rectus femoris quadriceps muscle), thus constituting a better indication of overall lower limb strength improvement.

Functional exercise capacity was measured using 6MWD in all five RCTs. This outcome improved significantly following 3 weeks of combined strength training and WBVT in two studies (Gloeckl et al. 2012, 2017). The improvements exceeded the minimum clinically important difference (MCID) of 14 m – 30 m (Bohannon & Crouch 2017) and may thus be considered clinically significant. Notably, the training programmes in these two studies were comparable between groups. These studies included both participants with severe to very severe COPD, supporting the potential value and feasibility of the modality in advanced disease stages. Similarly, Spielmanns et al. (2017a) found a statistically significant difference between WBVT and control favouring WBVT, but after a longer period (12 weeks). This study included patients with mild-to-severe COPD and results also exceeded the MCID. However, it again remains unknown whether the beneficial effect can truly be attributed to the added WBVT, or whether the entire intervention was more effective than calisthenics in this particular study. Two studies (Salhi et al. 2015; Spielmanns et al. 2017b) found no statistically significant between-group differences in FEC after 12 weeks (including for VO_2_ max). Salhi et al. (2015), however, did find a statistically significant intragroup improvement for both the WBVT and control groups. It thus seems that both a WBVT-and-squat-containing strength regime and a resistance programme without these components may be equally beneficial.

Despite the lack of evidence noted for improved isolated muscle strength, the observed improvements in FEC may be because of other neuromuscular adaptations associated with WBVT such as increased muscle power and the myostatic stretch reflex elicited by mechanical vibration (Salhi et al. 2015). Indeed, such changes – rather than muscle hypertrophy – have been associated with improvements in 6MWD (Gloeckl et al. 2015). A possible reason why two RCTs (Salhi et al. 2015; Spielmanns et al. 2017b) did not find a significant exercise capacity improvement in the WBVT group is that WBVT may not be specific enough to increase peripheral muscle strength or walking distance reliably, especially in higher functioning cohorts (Salhi et al. 2015). For example, participants in the studies conducted by both Salhi et al. (2015) and Spielmanns (2017b) had among the highest baseline 6MWD values and thus less room to show improvement compared to the studies that did show significant improvements.

In healthy elderly adults, positive, moderate correlations have been demonstrated between the strength and function of the respiratory muscles (measured using a vacuum manometer) and lower limb muscles (measured as knee extension peak torque), as well as functional capacity (measured using 6MWD) (Simões et al. 2010). In addition, WBVT combined with static squatting has been found to improve cardiorespiratory response (VO_2_, heart rate and oxygen saturation) in COPD (Lage et al. 2018) and respiratory muscle function and strength (inspiratory and expiratory pressure) (Pessoa et al. 2017). Tonic contractions generated by vibratory stimuli, along with isometric abdominal muscle contraction to maintain static positioning during training, are proposed mechanisms for the increased strength in expiratory muscles (Pessoa et al. 2017). In our review, pulmonary function was measured using FEV1 (% predicted) in three RCTs (Gloeckl et al. 2012; Salhi et al. 2015; Spielmanns et al. 2017b). None of these studies found significant differences in FEV1, either within or between groups. This suggests limited benefits of the intervention and control procedures on pulmonary function, at least in the short term (3–12 weeks). Chronic obstructive pulmonary disease level of severity across these studies ranged from mild (stage I) to very severe (stage IV); thus, this lack of improvement does not seem to be associated with COPD severity. It may be that in people with chronic respiratory diseases, neither WBVT nor lower limb strengthening (alone or combined) may be effective in improving lung function in a time period shorter than 3 months (Yang et al. 2016) – a finding also noted in studies in the cystic fibrosis population (Rietschel et al. 2008).

Although the our review found that leg press peak force improved significantly in the WBVT groups, no improvements were observed for knee extension peak torque. Considering the positive correlation previously observed between knee extension peak torque and lung function (Simões et al. 2010), this finding may perhaps be further supported for the proposed relationship.

Four RCTs assessed QoL, using three different measurement tools at 3 and 12 weeks. Although QoL improvements were noted at 12 weeks, these were not always of clinical relevance. For example, the significant (control group) improvements in CAT scores in the work of Spielmanns et al. (2017a) did not exceed the MCID of two points (Kon et al. 2014). This lack of clinical improvement may be because of the CAT not being specific enough to sense changes in exercise capacity, as only two of the eight questions related to physical activity (Spielmanns et al. 2017a). The improvements noted in Spielmanns et al. (2017b) for some individual CRDQ domains, on the contrary, did exceed the 0.5-point MCID (Schünemann et al. 2005) but should be interpreted cautiously because of the high participant dropout rate. Overall, given that the QoL measures may have lacked sensitivity and that different aspects of QoL were measured between studies, it is difficult to conclude whether adding WBVT to strength training is beneficial for improving QoL.

Overall, it is difficult to draw conclusions from our review regarding the best WBVT and strength training protocols to recommend to clinicians, as these varied vastly across studies. The intervention groups in all studies received a combination of WBVT and squat exercises to be performed on the vibrating platform. However, the type, number of squats and squatting positions differed between studies, as did the dosages. It has not been investigated yet if one squatting modality is superior to another, but it is speculated that a combination of both static and dynamic exercises may be beneficial to improve leg muscle function (Gloeckl et al. 2015). Our results also revealed wide variation in the type and dosage of additional exercise training prescribed to participants, and the specific WBVT device settings differed between studies. Current literature recommends aiming for a frequency of > 20 Hz for a side alternating platform, and < 35 Hz for a vertical platform, to improve muscle strength and function. These frequency ranges seem to increase neuromuscular activation of the lower limb muscles most effectively (Gloeckl et al. 2015). Vertical vibration may have a better long-term effect compared to side alternating motion (Yang et al. 2016) and WBVT duration sets of 30–180 s have been recommended depending on the specific training target. It is also suggested that WBVT amplitudes less than 6 mm do not result in significant treatment effects as compared to 6 mm – 10 mm, but the evidence remains insufficient regarding which amplitude is optimal (Yang et al. 2016).

Strengths of our review include a comprehensive and systematic search of eight scientific databases. The review was conducted according to a documented, stepwise process. Each step involved at least two independent authors, thus limiting potential errors because of cross-checking of information and increased objectivity. We identified five eligible RCTs (level II evidence) that were of a high methodological quality, although no studies were of excellent quality (i.e. > 8/10) (Armijo-Olivo et al. 2015). None of the studies were able to blind the participants, therapists or the assessors. Although inherent to the intervention employed, this could have led to observer or detection bias. Gloeckl et al. (2012) and Spielmanns et al. (2017a) did not apply concealed allocation, thus decreasing internal validity by causing selection bias, which may influence the type of treatment that the person will receive in the trial. Participant dropout (Spielmanns et al. 2017b) may have led to attrition bias and therefore potentially less reliable findings. The results of our review are limited by the fact that heterogeneity prohibited meta-analysis; however, performing meta-analyses of unsuitable data may have resulted in misleading findings. In addition, no conclusions can be drawn regarding long-term effects, as no studies had follow-up periods exceeding 12 weeks. The limited number of included studies and small sample sizes may affect the overall validity of our review results. Language bias is possible as only English papers were included. Finally, the fact that all studies were conducted in high-income countries limits generalisability to middle- and lower-income countries.

The clinical implications of this review’s findings should be considered carefully for physiotherapy practice, including the South African context. Given that South Africa is a high-middle income country with extreme levels of income inequality, and considering the high cost of WBVT devices, such equipment may fall outside of budgetary constraints for many South African clinics or physiotherapists – especially in rural settings. The current evidence should therefore be contemplated in terms of the specific goals of therapy and the needs of the person.

For example, if the goal of therapy is to improve muscle strength, the current evidence is insufficient to recommend WBVT over strength training alone, which is an effective modality for people with COPD (Barreiro & Gea 2015). Given that strength training without WBVT is more affordable, this is an important consideration for middle- or lower-income countries. On the contrary, if the primary goal is to improve FEC, combining WBVT with strength training may be considered as an even more effective modality than strength training alone, should resources allow. However, the substantial heterogeneity among the various studies makes it difficult to state guidelines for clinical practice and further research is required.

Future research should implement standardised exercise regimes and specifications regarding the settings and type of vibration plate. Similarly, standardising follow-up intervals and including long-term follow-up would provide greater consistency regarding results and the effect of combined therapy in the long-term management of a chronic condition such as COPD. Furthermore, greater attention should be paid to using valid, context-appropriate measurement tools that are specific enough to identify changes in COPD-related outcomes to ensure that results are valid.

## Conclusion

In conclusion, level II evidence suggests that short-term FEC may be improved more effectively by supplementing strength training with WBVT for adults with mild-to-severe COPD. Combining strength training with WBVT may not be more effective than strength training alone for improving isolated knee extension strength in adults with severe COPD. However, limited evidence (single study with a small sample size) suggests that leg press strength may improve after 12 weeks in those with mild-to-moderate COPD. Combined training was not found to be more effective than strength training alone for improving pulmonary function and evidence remain limited for QoL. Even though the current evidence suggests that if physiotherapists have the resources, and their primary goal of therapy is to improve FEC, they may consider combining strength training with WBVT, no recommendation can be made regarding optimal dosing and protocols. The results of our systematic review remain limited by the small number of available RCTs for inclusion and small sample sizes.
